# Association between serum CCL-18 and IL-23 concentrations and disease progression of chronic obstructive pulmonary disease

**DOI:** 10.1038/s41598-020-73903-6

**Published:** 2020-10-20

**Authors:** Biaoxue Rong, Tian Fu, Congxue Rong, Wen Liu, Kai Li, Hua Liu

**Affiliations:** 1 Department of Gerontology, The First Affiliated Hospital, Xi’an Medical University, 48 Fenghao West Road, Xi’an, 710077 China; 2 School of Clinical Medicine, Xi’an Medical University, Xi’an, China; 3 Department of Respiratory Medicine, Jining No. 1 People’s Hospital, Jining, China; 4 Comprehensive Medical Department, Zhangye Second People Hospital, Zhangye, China; 5 Department of Respiratory Medicine, Minqin County People’s Hospital, Minqin, China; 6 Department of Respiratory Medicine, Gansu Provincial Hospital, Lanzhou, China

**Keywords:** Cytokines, Diagnosis, Prognosis, Respiratory tract diseases

## Abstract

This study aimed to investigate the association between serum concentrations of chemokine (C–C Motif) ligand 18 (CCL-18) and interleukin 23 (IL-23) and clinical parameters of chronic obstructive pulmonary disease (COPD). The serum concentrations of CCL-18 and IL-23 were tested by enzyme linked immunosorbent assay (ELISA). The association between their concentrations and clinical parameters of COPD patients were analyzed by linear regression, logistic regression and ROC curve. The results showed that the serum concentrations of CCL-18 and IL-23 in COPD patients were increased compared with healthy people (*P* < 0.001) and that patients with acute exacerbation of COPD (AECOPD) had higher serum CCL-18 and IL-23 concentrations than stable patients (*P* < 0.001). Synergistic increase of CCL-18 and IL-23 in COPD patients was positively correlated with COPD patients' higher GOLD grade (*P* < 0.001), higher mMRC score (*P* < 0.001) and longer medical history (*P* < 0.001), but negatively correlated with the forced expiratory volume in one second (FEV1)/forced vital capacity (FVC) (*P* < 0.001) and FEV1% predicted (*P* < 0.001). The serum concentrations of CCL-18 and IL-23 were most related to the GOLD grade (OR = 2.764 for CCL-18 and OR = 4.215 for IL-23) and detection of both showed considerable sensitivity (72.57% for CCL-18 and 76.92% for IL-23) and specificity (92.50% for CCL-18 and 77.5% for IL-23) in identifying COPD. Increased serum concentrations of CCL-18 and IL-23 correlated with the disease progression of COPD and they could be used as biomarkers for disease evaluation of COPD.

## Introduction

Chronic obstructive pulmonary disease (COPD) is a disease characterized by persistent airflow limitation. Under the premise of individual genetic susceptibility factors, chronic inflammatory responses caused by various toxic stimuli to the airways and lungs will gradually cause diseases of small airways (obstructive bronchitis) and destruction of lung parenchyma (emphysema)^1^. China Pulmonary Health Study (CPHS) survey of 50,991 people shows that the prevalence of COPD among adults aged 20 and over is 8.6%, and as high as 13.7% over 40 years old. It is estimated that the number of COPD patients in China is close to one Billion^2^. Pulmonary inflammation, oxidative stress, protease and anti-protease imbalance play important roles in the pathogenesis of COPD^3^. Inflammatory cells, epithelial cells and other structural cells release a variety of inflammatory mediators, attract circulating inflammatory cells, amplify the inflammatory process, and induce changes in lung tissue structure^4,5^. Studies suggest that chronic airway inflammation in COPD involves multiple cytokines, which causes high secretion of mucus and obstruction of small airways, as well as damage to the lung parenchyma, and thus resulting in restricted airflow and excessive lung inflation^3–6^.

Chemokine (C–C motif) ligand 18 (CCL18) is a polypeptide composed of 69 amino acids with a molecular weight of 7.85 KDa, it is secreted protein that belongs to the intercrine beta (chemokine CC) family^4^. It is reported that CCL18 is highly expressed in the lung, lymph nodes, placenta, bone marrow, and dendritic cells, it is a chemotactic factor that attracts lymphocytes but not monocytes or granulocytes^7^. The increased CCL18 has been found to be involved in chronic airway inflammation, allergic pneumonia and pulmonary interstitial fibrosis^8^. Interleukin 23 (IL-23) cytokine is a heterodimeric cytokine consisting of the two subunits p19 and p40, it is an inducer of T helper cell 17 (Th17) cells and a component of IL-23/IL-17 immune pathway^7^. Studies show that IL-23 can induce naive CD4^+^ T cells to differentiate into highly pathogenic Th17/Thil-17 cells and the latter produces some inflammatory mediators and cytokines such as IL-6, IL-17 and tumor necrosis factor-α (TNF-α), thus involving in process of many diseases^7–11^. It has been showed that the elevated IL-23 in the body may trigger a waterfall cascade, leading to hyperplasia of airway mucus glands and excessive secretion of mucus, alveolar wall destruction and fibrosis, and airway structural reconstruction^7,10,11^. The aim of this study was to analyze the changes in the serum concentrations of CCL18 and IL-23 in patients with COPD, and explore the associations between their concentrations and clinical parameters of COPD.

## Results

### Serum concentrations of CCL-18 and IL-23 in COPD patients are higher than that in healthy people and the increase of serum CCL-18 and IL-23 correlates with clinical stage of COPD

**Figure 1:**
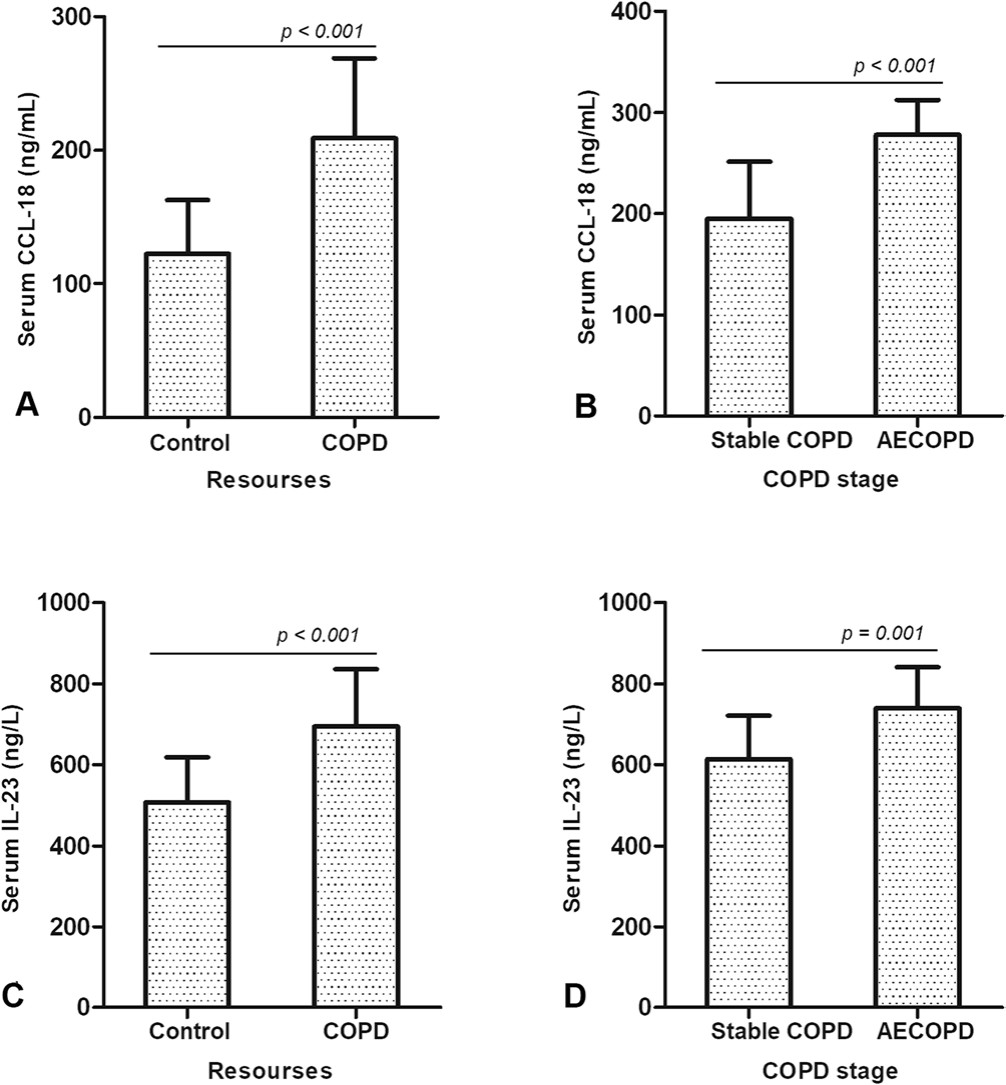
Changes of serum CCL-18 and IL-23 concentrations in COPD patients. (**A**) Patients with stable COPD showed higher serum CCL-18 concentrations compared with control group (*p* < 0.001). (**B**) The serum CCL-18 concentrations in AECOPD patients were higher than that in stable COPD patients (*p* < 0.001). (**C**) Patients with stable COPD showed high concentrations of serum IL-23 compared with control group (*p* < 0.001). (**D**) The serum IL-23 concentrations in AECOPD patients were higher than that in stable COPD patients (*p* = 0.001). COPD, chronic obstructive pulmonary disease; CCL-18, chemokine (C–C Motif) ligand 18; IL-23, interleukin 23; M ± SD, mean ± standard deviation.

Serum CCL-18 concentrations in COPD patients were higher (209.86 ± 62.64 ng/mL) than that in healthy people (122.53 ± 40.76 ng/mL) (*t* = 11.732, *p* < 0.001) (Supp. Table 1) (Fig. 1A). The CCL-18 concentrations in patients with acute exacerbation of COPD (AECOPD) (277.20 ± 35.76 ng/mL) showed a significant increase compared with stable COPD patients (194.49 ± 57.12 ng/mL) (*t* = − 8.438, *p* < 0.001) (Supp. Table 1) (Fig. 1B). The concentrations of serum IL-23 in COPD patients (659.89 ± 112.8 ng/L) were higher than that in healthy people (498.07 ± 82.02 ng/L) (*t* = 12.208, *p* < 0.001) (Supp. Table 2) (Fig. 1C). The concentrations of serum IL-23 in AECOPD patients (729.01 ± 102.31 ng/L) showed a significant increase compared with stable COPD patients (643.74 ± 108.53 ng/L) (*t* = − 3.416, *p* = 0.001) (Supp. Table 2) (Fig. 1D).

### High serum concentrations of CCL-18 are positively correlated with COPD patients' higher GOLD grade, higher mMRC score and longer clinical medical history

**Figure 2:**
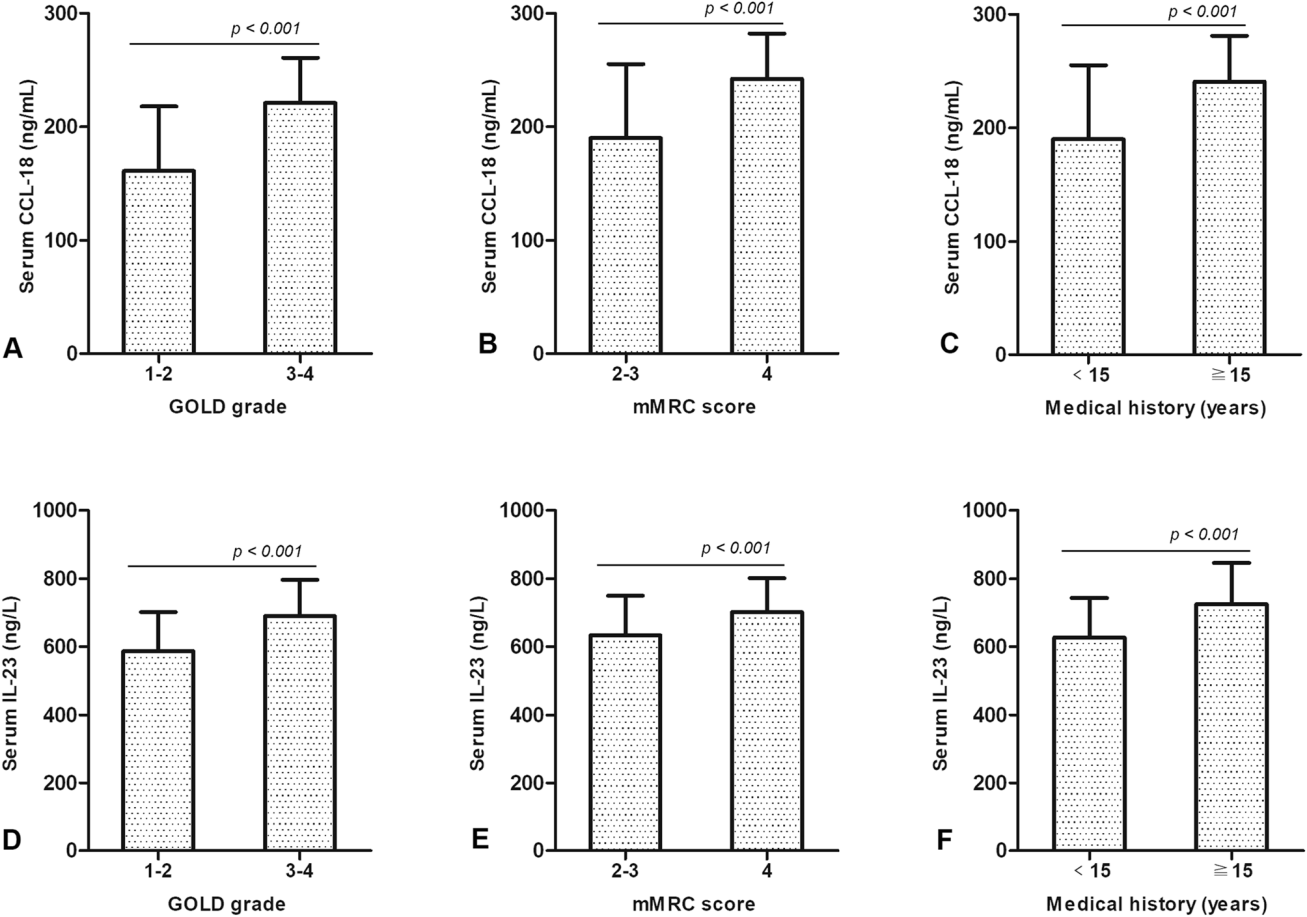
Relationships between clinical parameters and serum concentrations of CCL-18 and IL-23 in COPD patients. (**A**) The serum CCL-18 concentrations were higher in COPD patients of GOLD 3–4 than in those of GOLD 1–2 (*p* < 0.001). (**B**) The serum CCL-18 concentrations were higher in COPD patients with mMRC score of 4 than in those with mMRC score of 2–3 (*p* < 0.001). (**C**) The serum CCL-18 concentrations were higher in COPD patients with longer clinical history than in those with shorter clinical history (*p* < 0.001). (**D**) The serum IL-23 concentrations were higher in COPD patients of GOLD 3–4 than in those of GOLD 1–2 (*p* < 0.001). (**E**) The serum IL-23 concentrations were higher in COPD patients with mMRC score of 4 than in those with mMRC score of 2–3 (*p* < 0.001). (**F**) The serum IL-23 concentrations were higher in COPD patients with longer clinical history than in those with shorter clinical history (*p* < 0.001). COPD, chronic obstructive pulmonary disease; CCL-18, chemokine (C–C Motif) ligand 18; IL-23, interleukin 23; M ± SD, mean ± standard deviation; GOLD, Global Initiative for Chronic Obstructive Lung Disease; mMRC, modified british medical research council.

Serum CCL-18 concentrations were higher in COPD patients of GOLD 3–4 (221.36 ± 40.51 ng/mL), mMRC score of 4 (242.11 ± 40.85 ng/mL) and longer clinical medical history (241.91 ± 40.63 ng/mL) than that in those of GOLD 1–2 (161.05 ± 57.48 ng/mL) (*t* = − 5.896, *p* < 0.001) (Supp. Table 1) (Fig. 2A), mMRC score of 2–3 (190.05 ± 65.62 ng/mL) (*t* = − 5.198, *p* < 0.001) (Supp. Table 1) (Fig. 2B) and shorter medical history (190.90 ± 65.80 ng/mL) (*t* = − 5.093, *p* < 0.001) (Supp. Table 1) (Fig. 2C).

### High serum concentrations of IL-23 are positively correlated with COPD patients' higher GOLD grade, higher mMRC score and longer clinical medical history

Serum IL-23 concentrations were higher in COPD patients of GOLD 3–4 (689.05 ± 107.16 ng/L), mMRC score of 4 (701.38 ± 97.29 ng/L) and longer clinical medical history (724.76 ± 120.12 ng/L) than in those of GOLD 1–2 (586.78 ± 116.56 ng/L) (*t* = − 4.832, *p* < 0.001) (Supp. Table 2) (Fig. 2D), mMRC score of 2–3 (633.47 ± 117.79 ng/L) (*t* = − 3.651, *p* < 0.001) (Supp. Table 2) (Fig. 2E) and shorter medical history (626.98 ± 114.56 ng/L) (*t* = − 5.128, *p* < 0.001) (Supp. Table 2) (Fig. 2F).

### Serum CCL-18 and IL-23 concentrations have a positive correlation in COPD patients

*Pearson Correlation* analysis showed that serum concentrations of CCL-18 and IL-23 had a positive correlation in patients with COPD (*r* = 0.78, *p* < 0.001; *F* value of *ANOVA* = 172.077, *p* < 0.001) (Supp. Table 3) (Supp. Figure 1A,B). *Regression Coefficient* test suggested a correlation coefficient of 0.78 (*t* = 13.118, *p* < 0.001; 95% CI = 0.379 to 0.514). The linear regression equation was *^Y* = -*84.706* + *0.698X* (Supp. Table 3)*.*

### Serum CCL-18 concentrations are negatively correlated with the FEV1/FVC and FEV1% predicted in COPD patients

*Pearson Correlation* showed that there were negative correlations between the serum concentrations of CCL-18 and the value of forced expiratory volume in one second (FEV1)/forced vital capacity (FVC) (*r* = − 0.483, *p* < 0.001; *F* value of *ANOVA* = 27.113, *p* < 0.001; *t* value = − 0.508, *p* < 0.001; 95% CI = − 0.002 to − 0.001) (Supp. Table 3) (Supp. Figure 1C) and FEV1% predicted (*r* = − 0.502, *p* < 0.001; *F* value of *ANOVA* = 29.972, *p* < 0.001; *t* value = − 5.465, *p* < 0.001; 95% CI = − 0.002 to − 0.001) (Supp. Table 3) (Supp. Figure 1D) in COPD patients. The linear regression equations were *^Y* = *0.718*–*0.001X* and *^Y* = *0.698*–*0.001X* (Supp. Table 3)*.*

### Serum IL-23 concentrations are negatively correlated with the FEV1/FVC and FEV1% predicted in COPD patients

*Pearson Correlation* showed that there were negative correlations between the serum concentrations of IL-23 and the value of FEV1/FVC (*r* = − 0.421, *p* < 0.001; *F* value of *ANOVA* = 19.129, *P* < 0.001; *t* value = − 4.374, *p* < 0.001; 95% CI = − 0.001 to − 0.0001) (Supp. Table 3) (Supp. Figure 1E) and FEV1% predicted (*r* = − 0.536, *P* < 0.001; *F* value of *ANOVA* = 26.23, *p* < 0.001; *t* value = − 5.121, *p* < 0.001; 95% CI = − 0.001 to − 0.0001) (Supp. Table 3) (Supp. Figure 1F) in COPD patients. The linear regression equations were *^Y* = *0.834*–*0.001X* and *^Y* = *1.048*–*0.001X* (Supp. Table 3)*.*

### Sensitivity and specificity of serum CCL-18 and IL-23 to distinguish COPD from healthy people

**Figure 3:**
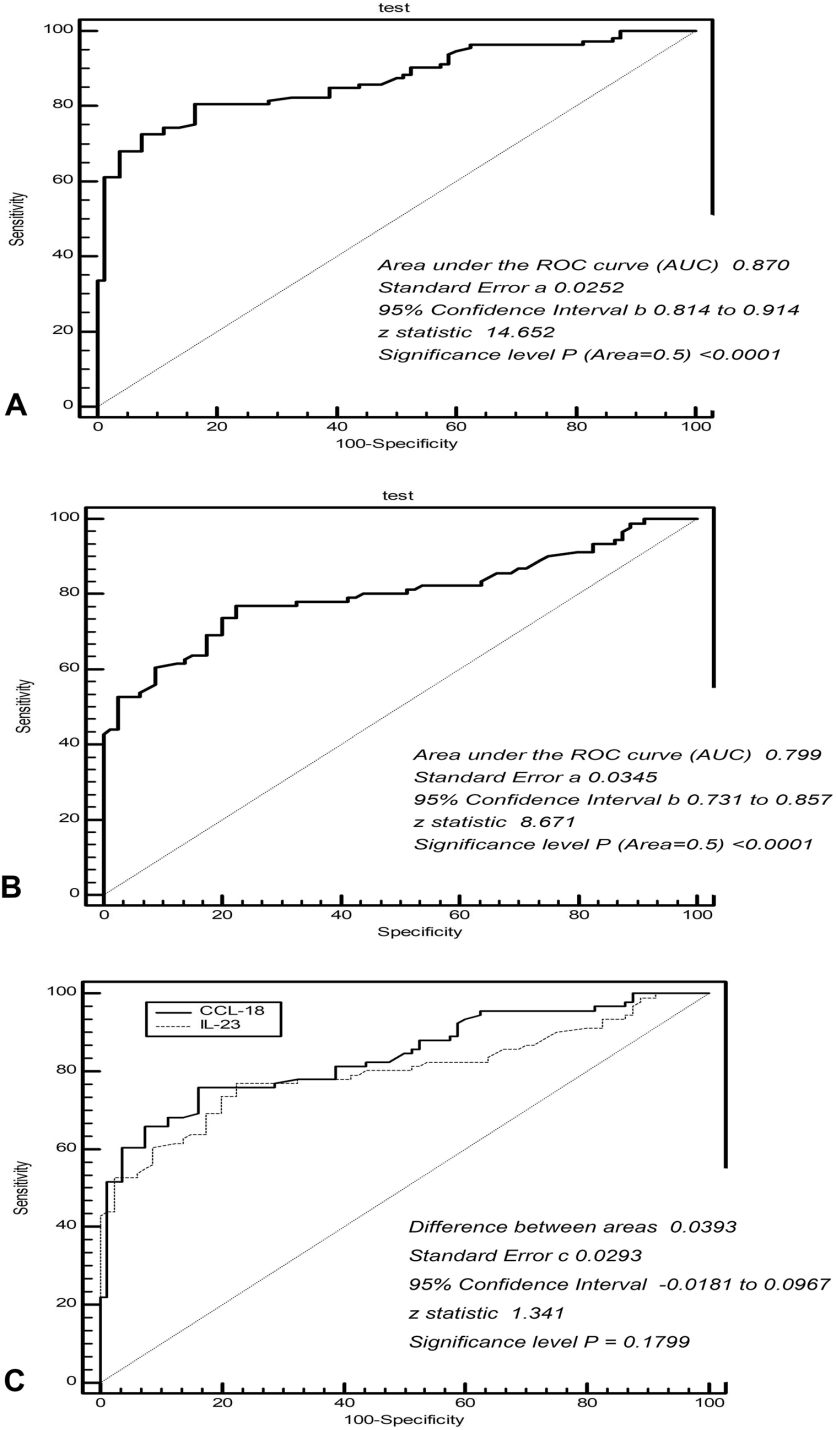
Sensitivity and specificity of serum CCL-18 and IL-23 concentrations to distinguish COPD from healthy people. (**A**) The ROC analysis showed that serum CCL-18 concentrations to distinguish COPD from healthy people had a moderate-high sensitivity and specificity (AUC = 0.870, *p* < 0.001). (**B**) To distinguish COPD from healthy people, serum IL-23 concentrations had a considerable sensitivity and specificity (AUC = 0.799, *p* < 0.001). (**C**) The sensitivity and specificity of serum CCL-18 and IL-23 to identify COPD did not show significant differences (difference between areas = 0.0393, *p* = 0.1799). CCL-18, chemokine (C–C Motif) ligand 18; IL-23, interleukin 23; AUC, area under the ROC curve; ROC, receiver operating characteristic curve.

The ROC analysis showed that a serum CCL-18 concentration of 168.3 ng/mL could be used as a cutoff value to distinguish COPD from healthy people (Supp. Table 4), responding a sensitivity of 72.57% (95% CI 63.4–80.5) and a specificity of 92.50% (95% CI 84.4–97.2) (area under the ROC curve (AUC) = 0.870, *Z* = 14.652, *p* < 0.0001) (Fig. 3A) (Supp. Figure 2A). In addition, a serum IL-23 concentration of 563.3 ng/L (Supp. Table 4) was found to be a cutoff value to distinguish COPD from healthy people (Supp. Table 4), suggesting that its sensitivity and specificity could reach 76.92% (95% CI 66.9–85.1) and 77.50% (95% CI 66.8–86.1) (AUC = 0.799, *Z* = 8.671, *p* < 0.001) (Fig. 3B) (Supp. Figure 2B). Comparing the CCL-18 and IL-23, the sensitivity and specificity of the two to identify COPD did not show a significant differences (difference between areas = 0.0393, *Z* = 1.341, *p* = 0.1799) (Fig. 3C) (Supp. Figure 2C).

### Logistic regression analysis between serum CCL-18 and IL-23 concentrations and clinical parameters of COPD

*Logistic Regression* showed that the variables that were eventually included in the regression equation were GOLD grade (*p* = 0.006, OR = 2.764, 95% CI = 1.334 to 5.728) and clinical medical history (*p* = 0.008, OR = 7.358, 95% CI = 0.784 to 70.971), suggesting that these two factors had a greater impact on serum concentrations of CCL-18 (*Logistic Regression Equation*: *P* = 1/[1 + e ^−(−4.193+1.017GOLD grade+2.009medical history)^]) (Supp. Table 5). In addition, the GOLD grade (*p* < 0.001, OR = 4.215, 95% CI = 2.052 to 8.656) seemed to directly affect the serum concentrations of IL-23 (*Logistic Regression Equation*: P = 1/[1 + e^−(−2.153+1.439GOLD grade)^]) (Supp. Table 6).

## Discussion

It is generally believed that COPD is characterized by chronic inflammation of the airways, lung parenchyma, and pulmonary blood vessels. Alveolar macrophages, T lymphocytes (especially CD) and neutrophils are increased in different parts of the lung^3,12,13^. It has been found that the activation of various inflammatory mediators and cytokines runs through the entire chronic inflammatory process, including leukotriene B4 (LTB4), IL-8, tumor necrosis factor-α (TNF-α), and other mediators^3,9,12–15^. In this study, we tested the changes in serum CCL18 and IL-23 concentrations in patients with COPD and found that the serum CCL-18 and IL-23 concentrations in COPD patients were higher than those in healthy people, which means that these two proteins are related to the occurrence and development of COPD. In addition, we found that their concentrations in AECOPD were significantly higher than that in stable COPD, indicating that they can respond quickly to the disease progression of COPD. Studies have shown that CCL-18 can induce the aggregation of inflammatory cells, participate in the activation of many inflammatory signaling pathways, and thus involve in the enlargement and chronicity of inflammation^16–18^. IL-23 has been reported to be an important chaperone in the proliferation and stability of Th17 cells, and it promotes the production of IL-17A, IL-17F, IL-22, thus involves in the development of inflammatory diseases^10,11^. IL-23 mediates lung inflammation and emphysema formation through the IL-23/IL-17 pathway^19^. A potential mechanism by which erythromycin reduces airway inflammation in patients with COPD may involve inhibition of IL-17/IL-23-mediated signaling pathways^20^.

The grade of COPD severity (GOLD grading) is based on two clinical indices, FEV1/FVC and FEV1% predicted, which are sensitive indicators for evaluating airflow limitation^21,22^. The GOLD recommends the mMRC Dyspnea Scale as a tool for respiratory function assessment^23^. In our study, we found that serum concentrations of CCL-18 and IL-23 were positively correlated with COPD patients’ higher GOLD grade and higher mMRC score, which means that the increase of the two is related to the deterioration of COPD. The persistently high levels of CCL-18 in the lung can upregulate the expressions of TNF-α, INF-γ, matrix metalloproteinase (MMP)-2 and MMP-9, thereby aggravating the pulmonary inflammation response^24^. Increased CCL-18 in COPD is significantly correlated with the proportion of FEV1 decline and the risk of future acute exacerbations^17,18^. Interestingly, we found a positive linear association between the serum CCL-18 and IL-23 concentrations in patients with COPD, which suggests that they may exert a synergistic effect in the progression of COPD. We also noticed that the serum CCL-18 and IL-23 concentrations were negatively correlated with the FEV1/FVC and FEV1% predicted in COPD patients, indicating that the inflammatory response mediated by CCL-18 and IL-23 is closely related to the degree of lung function damage in COPD patients. A previous study shows that the CCL-18 is highly expressed in the lungs, and exerts the chemotactic effects on lymphocytes, but has also been shown to stimulate fibrinogen activity and collagen production in lung fibroblasts^18^. Respiratory airflow limitation in COPD patients is associated with increased mucus secretion in the airways, and excessive mucus secretion is associated with decreased FEV1. IL-23 can stimulate bronchial epithelial goblet cells and submucosal glands to increase mucus secretion^9–11^, which has been shown to be facilitated by elastase-induced pulmonary inflammation and emphysema formation mediated by the IL-23/IL-17 pathway^7–11,19^.

The ROC curve analysis in our study showed that CCL-18 (sensitivity of 72.57% and specificity of 92.50%) and IL-23 (sensitivity of 76.92% and specificity of 77.50%) could be used to distinguish COPD from healthy people. The *Logistic Regression* analysis in our study showed that the GOLD grade (OR = 2.764) and clinical medical history (OR = 7.358) were primarily responsible for the increase in CCL-18 concentrations and that the GOLD grade (OR = 4.215) affected the serum concentrations of IL-23. Our findings indicate that CCL-18 and IL-23 participate in the inflammatory process of COPD and are correlated with irreversible airway inflammation and airflow restriction, so they may be used as inflammatory markers for disease evaluation and efficacy evaluation of COPD. We speculate that there may be a vicious cycle of positive feedback regulation. The deterioration of COPD disease promotes the release of pro-inflammatory factors such as CCL-18 and IL-23 in the body, leading to accumulation of macrophages and neutrophils in the lung, further aggravating COPD chronic inflammation of the airway, vice versa^7,11,16–19^. Our findings will provide some references for further explanation of the etiology and pathogenesis of COPD.

The shortcomings of this study may have the following points. These patients included in the study may not reflect the characteristics of different geographical conditions and ethnicities. This study is only a non-intervention observational study and did not perform testing for changes on CCL-18 and IL-23 in patients with COPD after any treatment. The study did not focus on the molecular mechanisms of CCL-18 and IL-23 changes in COPD. The subjects of this study are mainly stable COPD, and AECOPD is only a small part of it. The study would have been more robust had it considered “stable COPD” and “AECOPD” as two separate groups. In future research, we should pay attention to the above topics. The conclusions of this study suggest that CCL-18 and IL-23 are involved in the occurrence and development of COPD, and have certain clinical value in assessing the severity of the disease and judging the prognosis of patients. They are expected to become biomarkers of COPD, and interventions for them are expected to become new therapeutic targets for COPD.

## Conclusion

COPD patients had higher serum CCL-18 and IL-23 concentrations than healthy subjects, and the patients with AECOPD showed higher concentrations than those with stable patients. Increased CCL-18 and IL-23 were positively correlated with COPD patients' higher GOLD grade, higher mMRC score, but negatively related to the FEV1/FVC and FEV1% predicted. The testing of serum concentrations for CCL-18 and IL-23 contributed to diagnosis and evaluation of COPD, and their concentrations were most affected by the GOLD grading.

## Methods

### Ethics statement

This study was a retrospective observational study and did not involve any form of therapeutic intervention. Written informed consent was obtained from all patients recruited into the study. The study was approved by the ethics committee of the Research Ethics Committees of research institutes (Jining NO.1 People's Hospital, Jining, China; Zhangye Second People Hospital, Zhangye, China; Minqin County People's Hospital, Minqin, China; Gansu Provincial Hospital, Lanzhou, China; First Affiliated Hospital, Xi'an Medical University, Xi'an, China). We confirm that all methods were performed in accordance with the relevant guidelines and regulations.

### Patients

From January 2018 to December 2019, 113 COPD patients and 80 healthy controls from 5 hospitals (Jining NO.1 People's Hospital, Jining, China; Zhangye Second People Hospital, Zhangye, China; Minqin County People's Hospital, Minqin, China; Gansu Provincial Hospital, Lanzhou, China; First Affiliated Hospital, Xi'an Medical University, Xi'an, China) were included in this observational study. The order in which patients and healthy controls were enrolled in the group was determined based on their visit time to each hospital. There were 92 cases in stable COPD group and 21 cases in AECOPD group, including 61 males and 52 females, with an average age of (68.85 ± 12.4) years. The 80 healthy controls were from the outpatient medical examination department, including 35 males and 45 females with an average age of (69.43 ± 11.7) years. There were no significant differences in demographic characteristics such as gender, age, and smoking between the two groups (*p* > 0.05) (Table 1).

**Table 1:** Demographic characteristics of included populations (COPD = 113; Control = 80).

Items	Group	COPD (N, %)	Control (N, %)	P value
**Gender**				
	Male	61 (54)	35 (43.8)	> 0.05
	Female	52 (46)	45 (56.2)	
**Age (years)**				
	≤ 65	43 (38.1)	28 (35)	> 0.05
	> 65	70 (60.9)	52 (65)	
**Smoking**^**a**^				
	Yes	52 (46)	25 (31.3)	> 0.05
	No	61 (54)	55 (68.7)	
**BMI**				
	≥ 18.5 ≤ 24.9	70 (61.9)		
	≥ 25 ≤ 30	43 (38.1)		
**COPD stage**				
	Stable	92 (81.4)		
	Acute exacerbation	21 (18.6)		
**GOLD grade**				
	1–2	41 (36.3)		
	3–4	51 (45.1)		
	Unavailable	21 (18.6)		
**mMRC score**				
	2–3	70 (61.9)		
	4	43 (38.1)		
**Medical history (years)**^**b**^				
	> 15	71 (62.8)		
	≥ 15	42 (37.2)		

*BMI* body mass index, *mMRC* modified british medical research council for dyspnea scale for symptom classification of COPD, *COPD* chronic obstructive pulmonary disease, *GOLD* global initiative for chronic obstructive lung disease.

^a^"Yes" in the smoking category refers to current smokers; "No" refers to those who have never smoked or have quit smoking for more than 3 months. GOLD grade: 1 = the forced expiratory volume in one second (FEV1) % predicted is more or equal to 80%, 2 = the FEV1% predicted is more or equal to 50%, but less than 80%, 3 = the FEV1% predicted is more or equal to 30%, but less than 50%, and 4 = the FEV1% predicted is less than 30%.

^b^"Medical history" refers to the time span (years) from the diagnosis of COPD to the patient being enrolled in this study.

### Pulmonary function test

All stable COPD patients and healthy controls received lung function test before collecting blood samples. AECOPD patients were unable to cooperate with this test because of severity of the disease, the test was not required mandatorily (the reports of previous pulmonary function testing are needed to confirm the diagnosis of COPD). Pulmonary function test was performed using a pulmonary function meter, the forced expiratory volume in one second (FEV1)/forced vital capacity (FVC) and FEV1% predicted were recorded. The test was performed three times at different times; an average value was taken for statistical analysis^25^.

### Diagnosis and clinical staging of COPD

Diagnosis for COPD patients based strictly on the Global Chronic Obstructive Pulmonary Disease Initiative (GOLD)^21,25^: FEV1/FVC is less than 70% and FEV1 < 80% predicted (strictly FEV1 after antispasmodic drugs) and airflow obstructive diseases with known specific pathology such as cystic fibrosis, occlusive bronchitis, etc., need to be excluded^22^. AECOPD refers to the worsening of cough, shortness of breath, wheezing, increased purulent or mucopurulent sputum volume and fever, etc.; stable COPD means that the above symptoms are stable or relatively mild^22,25^. All healthy controls underwent pulmonary function tests, and the COPD was excluded.

### GOLD grading for COPD severity

Stable COPD patients' severity is defined according to the results of lung function test according to the GOLD criteria^22^. GOLD-1: FEV1/FVC < 70% and FEV1 ≥ 80% predicted; GOLD-2: FEV1/FVC < 70% and 50% ≤ FEV1 < 80% predicted; GOLD-3: FEV1/FVC < 70% and 30% ≤ FEV1 < 50% predicted; GOLD-4: FEV1/FVC < 70% and FEV1 < 30% predicted.

### Inclusion and exclusion criteria for COPD patients

Inclusion criteria are as follows: (1) met diagnostic criteria for COPD^22,25^; (2) had a pulmonary function test report within 3 days (AECOPD patients did not make this requirement); (3) stable COPD patients had a stable period of more than 2 weeks; and (4) no treatment with oxygen, antibiotics, glucocorticoids, and theophylline within the last 1 month. Exclusion criteria are as follows: (1) treated with immunosuppressive drugs in the past month; (2) other airflow-limited diseases, including bronchiectasis, tuberculosis, lung cystic fibrosis and tumors and so on; (3) with severe heart, brain, liver, kidney, hematopoietic, endocrine system and skin diseases; (4) with mental illness, mental disorder, and cognitive impairment; (5) except for AECOPD, patients with stable COPD must not have respiratory infections, and (6) infectious diseases other than the respiratory system.

### Specimen preparation

Early morning fasting, 5 mL of venous blood without anticoagulants was collected from the elbow vein of the subjects, which was stored in a centrifuge tube and left to coagulate at room temperature. The coagulated blood was equilibrated and centrifuged (3000 rpm, 5 to 10 min) to obtain supernatants. The supernatants were carefully sucked out of the centrifuge tube and put into new EP tubes, and stored in a − 20 °C refrigerator.

### Enzyme-linked immunosorbent assay (ELISA)

The concentrations of CCL-18 (Jinma Biotechnology Co., Ltd., Shanghai, China) and IL-23 (Fangchen Biotechnology Co., Ltd., Beijing, China) in serum were measured by sandwich-type ELISA. Briefly, the microtiter plates were coated with purified CCL-18 and IL-23 antibodies to make solid-phase antibodies. The smples were sequentially added to the microwells coated with the monoclonal antibody, and combined with HRP-labeled CCL-18 and IL-23 antibodies to form an antibody-antigen-enzyme-labeled antibody complex. After thorough washing, the substrate TMB was used to develop the color. TMB converted to blue under the catalysis of HRP enzyme, and turned into the final yellow under the action of acid. The absorbance (OD value) was measured with a microplate reader at a wavelength of 450 nm, and the concentrations of CCL-18 and IL-23 in the sample were calculated by a standard curve. Intra-group variation analysis: the same sample was tested for 3 times on the same microplate; between-group variation analysis: the same sample was tested repeatedly three times at different times; and data analysis showed that the variation did not affect the detection results.

### Modified british medical research council (mMRC) score

Dyspnea of patients was evaluated by the modified british medical research council (mMRC) scale^23^. Grade 0, I only get breathless with strenuous exercise; Grade 1, I get short of breath when hurrying on level ground or walking up a slight hill; Grade 2, on level ground, I walk slower than people of the same age because of breathlessness, or I have to stop for breath when walking at my own pace on the level; Grade 3, I stop for breath after walking about 100 yards or after a few minutes on level ground; Grade 4, I am too breathless to leave the house or I am breathless when dressing.

### Statistics of data

The statistical ideas applied are as follows: (1) the statistical software used in the analysis was the SPSS 20.0; (2) the measurement data are expressed by mean ± standard deviation (M ± SD) and the comparison of the two sample means was analyzed by *t* test; (3) before the analysis, the *Levene’ s Test for Equality of Variances* test for the data was performed; Student's t test was used to analyze normally distributed data with unknown population standard deviation σ, and infer the probability of the difference through t distribution theory; the results of t test was taken for uniform variance and the corrected result of t test was taken for uneven variance; (4) the percentage was used to represent the count data, and the chi-square test was used to compare the two sample rates; (5) *Pearson Correlation Test* was used to analyze the relationship between the indicators; (6) receiver operating characteristic curve (ROC) analysis was used to determine the cutoff value, sensitivity, and specificity of observations; the ROC curve analysis was applied in our study design according to The Standards for Reporting of Diagnostic Accuracy (STARD) Initiative guidelines^26^; and (7) *P* < 0.05 was considered statistically significant.

## Supplementary information

Supplementary figures.

Supplementary tables.

## Data Availability

The datasets generated during and/or analysed during the current study are available from the corresponding author on reasonable request.
