# Supplementary material for: Association between serum CCL-18 and IL-23 concentrations and disease progression of chronic obstructive pulmonary disease
Source: Sci Rep. 2020 Oct 20;10:17756. doi: 10.1038/s41598-020-73903-6 (PMC7576212; doi:10.1038/s41598-020-73903-6)
Supplement: Supplementary file 1 — Supplementary figures. [file 41598_2020_73903_MOESM1_ESM.doc]

***Original Research***

**Association between serum CCL-18 and IL-23 concentrations and disease progression of chronic obstructive pulmonary disease**

**Short title: Association between CCL-18 and IL-23 and COPD**

**Biaoxue Rong^1,2*^, Tian Fu^3^, Congxue Rong^4^, Wen Liu^5^, Kai Li^2^** **& Hua Liu^6^**

^1^Department of Gerontology, The First Affiliated Hospital, Xi’an Medical University, Xi’an, China

^2^School of Clinical Medicine, Xi’an Medical University, Xi’an, China

^3^Department of Respiratory Medicine, Jining NO.1 People's Hospital, Jining, China

^4^Comprehensive Medical Department, Zhangye Second People Hospital, Zhangye, China

^5^Department of Respiratory Medicine, Minqin County People's Hospital, Minqin, China

^6^Department of Respiratory Medicine, Gansu Provincial Hospital, Lanzhou, China

^★^Corresponding author: Biaoxue Rong, Department of Gerontology, First Affiliated Hospital, Xi’an Medical University, Xi’an, China, 48 Fenghao West Road, Xi’an 710077, China. Tel: +86 029-87679300; E-mail: [research568rbx@yeah.net](mailto:research568rbx@yeah.net)

Tian Fu [aifutian@sohu.com](mailto:aifutian@sohu.com)

Congxue Rong 281985167@qq.com

Wen Liu [1872359048@qq.com](mailto:1872359048@qq.com)

Kai Li rbx3666610@163.com

Hua Liu 15349260656@139.com


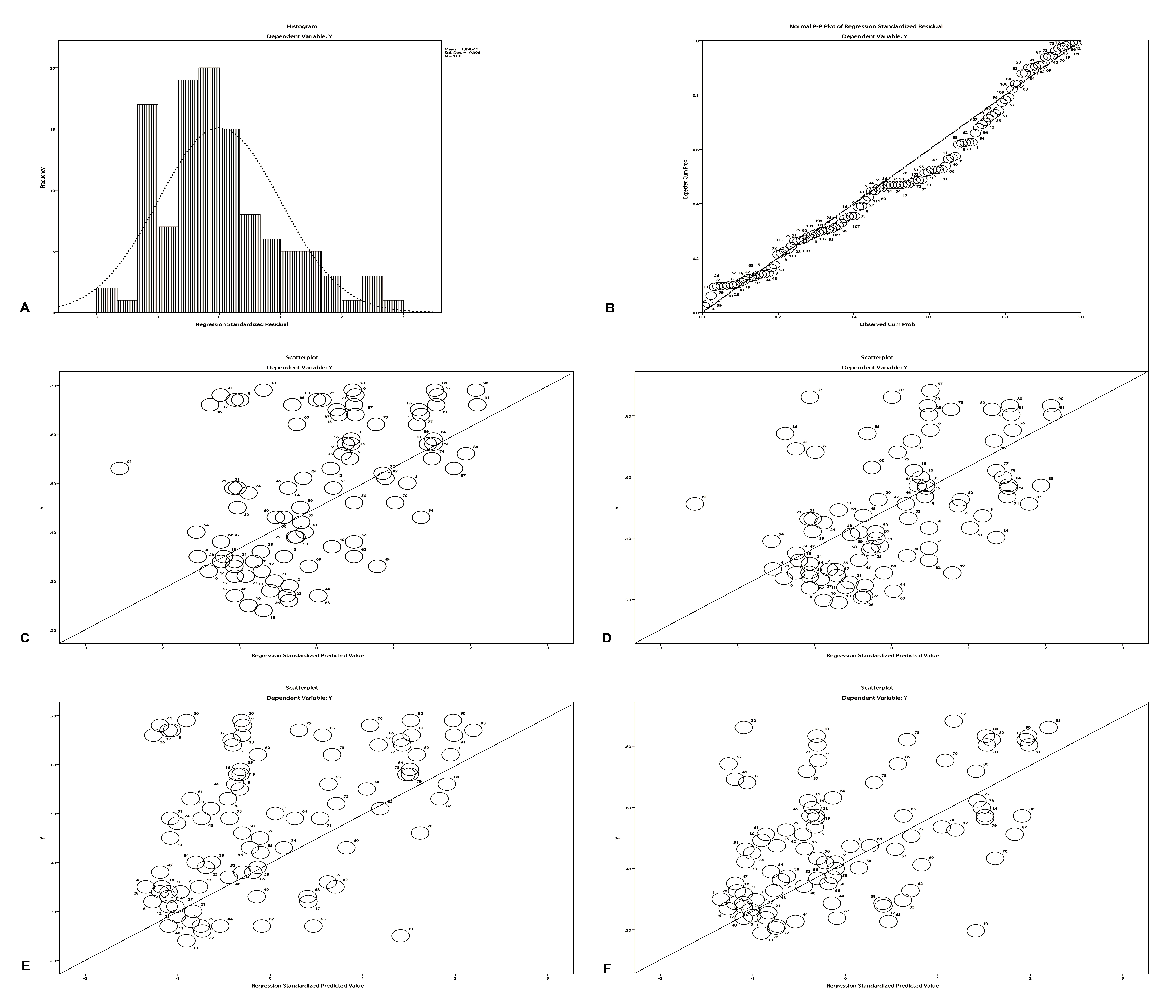


**Supp Figure 1.** Relationships between serum concentrations of CCL-18 and IL-23 and pulmonary function in stable COPD. (**A** and **B**) *Pearson Correlation* analysis showed that the serum concentrations of CCL-18 and IL-23 had a positive correlation (*r*=0.78, *p*<0.001). (**C**) The dependent variable was approximately linear with the standardized predictive value and the cumulative probability plot of the observations showed that the two variables had a feature of normal distribution, suggesting there were negative correlations between the serum concentrations of CCL-18 and the value of FEV1/FVC (*r*= -0.483, *p*<0.001). (**D**) The dependent variable was approximately linear with the standardized predictive value and the cumulative probability plot of the observations showed that the two variables had a feature of normal distribution, suggesting there were negative correlations between the serum concentrations of CCL-18 and the value of FEV1% predicted (*r*= -0.502, *p*<0.001). (**E**) The dependent variable was approximately linear with the standardized predictive value and the cumulative probability plot of the observations showed that the two variables had a feature of normal distribution, suggesting there were negative correlations between the serum concentrations of IL-23 and the value of FEV1/FVC (*r*= -0.421, *p*<0.001). (**F**) The dependent variable was approximately linear with the standardized predictive value and the cumulative probability plot of the observations showed that the two variables had a feature of normal distribution, suggesting there were negative correlations between the serum concentrations of IL-23 and the value of FEV1% predicted (*r*= -0.536, *p*<0.001). CCL-18, chemokine (C-C Motif) ligand 18; IL-23, interleukin 23; FEV1, forced expiratory volume in one second; FVC, forced vital capacity.


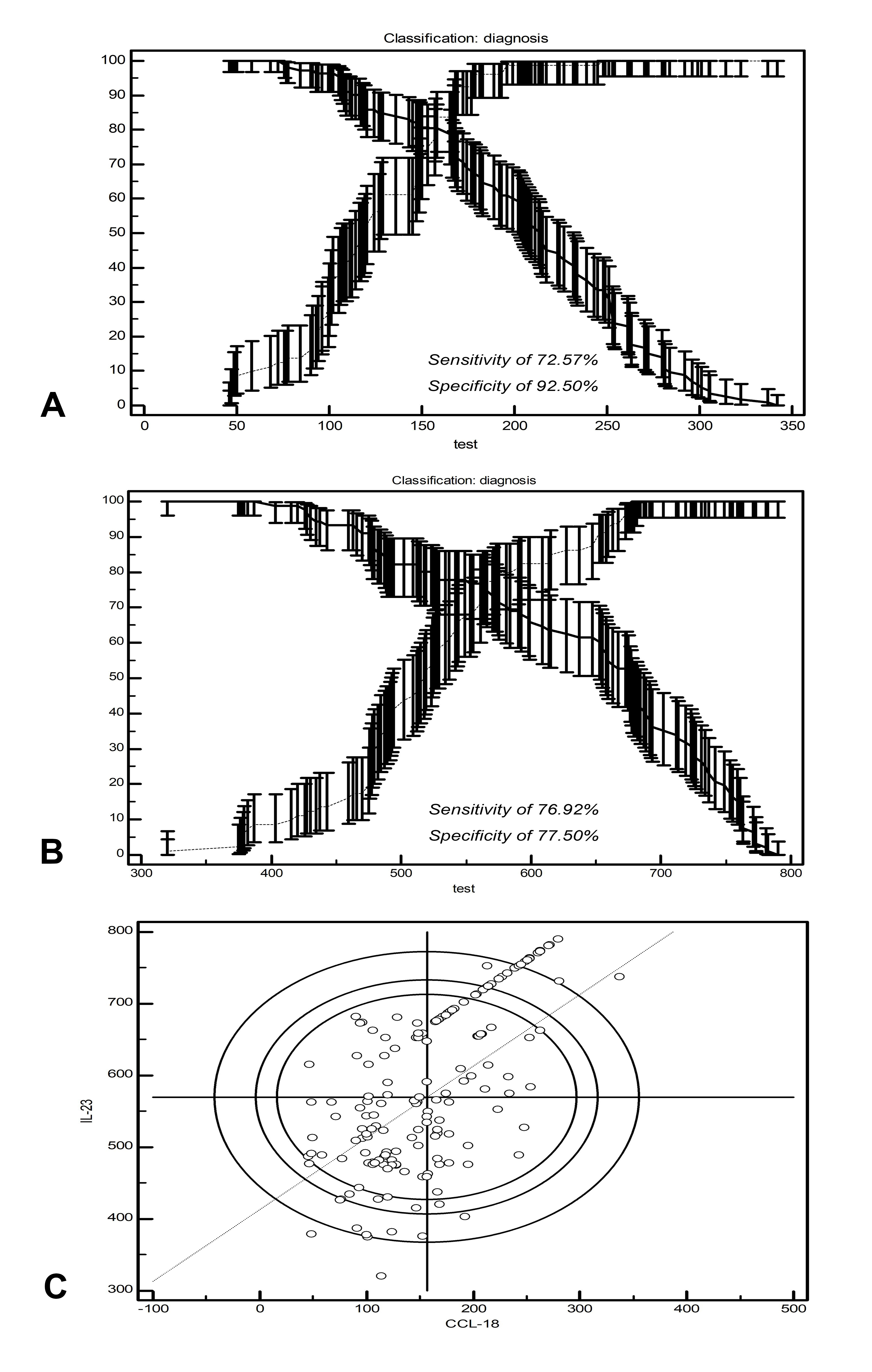


**Supp Figure 2.** Sensitivity and specificity of serum CCL-18 and IL-23 concentrations to distinguish COPD from healthy people. (**A**) The ROC analysis showed that the sensitivity and specificity of serum CCL-18 concentrations to distinguish COPD from healthy people were 72.57% and 92.50%. (**B**) To distinguish COPD from healthy people, the sensitivity and specificity of serum IL-23 concentrations could reach 76.92% and 77.50%. (**C**) Comparing the serum concentrations of CCL-18 and IL-23, *Youden* index showed a good inspection performance. CCL-18, chemokine (C-C Motif) ligand 18; IL-23, interleukin 23; ROC, receiver operating characteristic curve.
