# Supplementary material for: Association between serum CCL-18 and IL-23 concentrations and disease progression of chronic obstructive pulmonary disease
Source: Sci Rep. 2020 Oct 20;10:17756. doi: 10.1038/s41598-020-73903-6 (PMC7576212; doi:10.1038/s41598-020-73903-6)
Supplement: Supplementary file 2 — Supplementary tables. [file 41598_2020_73903_MOESM2_ESM.doc]

| Parameter | Group | N | Statistical analysis for serum concentration of CCL-18 | | | | | |
| --- | --- | --- | --- | --- | --- | --- | --- | --- |
|  |  |  | Value  (M±SD ng/mL) | Degree of  freedom | Statistical  value | *p* value | 95% Confidence Interval | |
|  |  |  |  |  |  |  | Lower | Upper |
| Resourses |  |  |  |  |  |  |  |  |
|  | Control | 80 | 122.53±40.76 | 189.65 | 11.732^▲^ | <0.001 | 75.58 | 103.53 |
|  | COPD | 113 | 209.86±62.64 |  |  |  |  |  |
| COPD stage |  |  |  |  |  |  |  |  |
|  | Stable | 92 | 194.49±57.12 | 101.55 | ﹣8.438^▲^ | <0.001 | ﹣102.49 | ﹣62.97 |
|  | AECOPD | 21 | 277.20±35.76 |  |  |  |  |  |
| Gender |  |  |  |  |  |  |  |  |
|  | Male | 61 | 209.95±63.39 | 111 | 0.018 | 0.995 | ﹣23.29 | 23.72 |
|  | Female | 52 | 206.46±62.36 |  |  |  |  |  |
| Ages |  |  |  |  |  |  |  |  |
|  | ≦65 | 43 | 190.04±65.98 | 111 | ﹣1.716 | 0.063 | ﹣56.26 | ﹣7.79 |
|  | ﹥65 | 70 | 212.03±57.66 |  |  |  |  |  |
| Smoking^◆^ |  |  |  |  |  |  |  |  |
|  | No | 61 | 206.78±59.26 | 111 | 0.561 | 0.581 | ﹣30.18 | 17.06 |
|  | Yes | 52 | 213.46±66.78 |  |  |  |  |  |
| GOLD grade^●^ |  |  |  |  |  |  |  |  |
|  | 1-2 | 41 | 161.05±57.48 | 90 | ﹣5.896 | <0.001 | ﹣80.64 | ﹣39.98 |
|  | 3-4 | 51 | 221.36±40.51 |  |  |  |  |  |
| mMRC score |  |  |  |  |  |  |  |  |
|  | 2-3 | 70 | 190.05±65.62 | 110.97 | ﹣5.198^▲^ | <0.001 | ﹣71.91 | ﹣32.22 |
|  | 4 | 43 | 242.11±40.85 |  |  |  |  |  |
| Medical history (years) |  |  |  |  |  |  |  |  |
|  | ﹤15 | 71 | 190.90±65.80 | 111 | ﹣5.093 | <0.001 | ﹣70.85 | ﹣31.16 |
|  | ≧15 | 42 | 241.91±40.63 |  |  |  |  |  |

Title: Association between serum CCL-18 and IL-23 concentrations and disease progression of chronic obstructive pulmonary disease

Author list: Biaoxue Rong^1,2*^, Tian Fu^3^, Congxue Rong^4^, Wen Liu^5^, Kai Li^2^ & Hua Liu^6^

**Supp Table 1.** Relationship between clinical parameters and serum concentration of CCL-18 in COPD patients (N﹦113). ^●^, twenty-one patients with acute exacerbation of COPD were unable to complete pulmonary function tests and did not participate in the statistical analysis of GOLD grade; ^▲^, the Levene’ s Test for Equality of Variances indicates that the variance is uneven, so the correction result of the t test is taken; ^◆^, "Yes" in the smoking category refers to current smokers. "No" refers to those who have never smoked or have quit smoking for more than 3 months. GOLD grade: 1﹦the forced expiratory volume in one second (FEV1) % predicted is more or equal to 80%, 2﹦the FEV1 % predicted is more or equal to 50%, but less than 80%, 3﹦the FEV1 % predicted is more or equal to 30%, but less than 50%, and 4﹦the FEV1 % predicted is less than 30%; mMRC, modified british medical research council for dyspnea scale for symptom classification of COPD. N, number; COPD, chronic obstructive pulmonary disease; AECOPD, acute exacerbation of COPD; CCL-18, chemokine (C-C Motif) ligand 18; M±SD, mean ± standard deviation; GOLD, Global Initiative for Chronic Obstructive Lung Disease; mMRC, modified british medical research council.

| Parameter | Group | N | Statistical analysis for serum concentration of IL-23 | | | | | |
| --- | --- | --- | --- | --- | --- | --- | --- | --- |
|  |  |  | Value  (M±SD ng/L) | Degree of  freedom | Statistical  value | *p* value | 95% Confidence Interval | |
|  |  |  |  |  |  |  | Lower | Upper |
| Resourses |  |  |  |  |  |  |  |  |
|  | Control | 80 | 498.07±82.02 | 183.269 | 12.208^▲^ | <0.001 | 126.97 | 175.93 |
|  | COPD | 113 | 659.89±112.8 |  |  |  |  |  |
| COPD stage |  |  |  |  |  |  |  |  |
|  | Stable | 92 | 643.74±108.53 | 111 | ﹣3.416 | 0.001 | ﹣136.14 | ﹣35.97 |
|  | AECOPD | 21 | 729.01±102.31 |  |  |  |  |  |
| Gender |  |  |  |  |  |  |  |  |
|  | Male | 61 | 668.74±108.53 | 111 | 0.967 | 0.334 | ﹣21.01 | 60.81 |
|  | Female | 52 | 648.01±110.37 |  |  |  |  |  |
| Ages |  |  |  |  |  |  |  |  |
|  | ≦65 | 43 | 629.11±123.56 | 111 | ﹣2.291 | 0.052 | ﹣92.14 | ﹣7.31 |
|  | ﹥65 | 70 | 655.91±97.81 |  |  |  |  |  |
| Smoking^◆^ |  |  |  |  |  |  |  |  |
|  | No | 61 | 673.17±129.08 | 111 | ﹣0.344 | 0.731 | ﹣39.85 | 28.05 |
|  | Yes | 52 | 649.17±109.14 |  |  |  |  |  |
| GOLD grade^●^ |  |  |  |  |  |  |  |  |
|  | 1-2 | 41 | 586.78±116.56 | 66.757 | ﹣4.832^▲^ | <0.001 | ﹣144.35 | ﹣59.93 |
|  | 3-4 | 51 | 689.05±107.16 |  |  |  |  |  |
| mMRC score |  |  |  |  |  |  |  |  |
|  | 2-3 | 70 | 633.47±117.79 | 109.746 | ﹣3.651^▲^ | <0.001 | ﹣104.51 | ﹣30.88 |
|  | 4 | 43 | 701.38±97.29 |  |  |  |  |  |
| Medical history (years) |  |  |  |  |  |  |  |  |
|  | ﹤15 | 71 | 626.98±114.56 | 110.822 | ﹣5.128 | <0.001 | ﹣124.56 | ﹣54.87 |
|  | ≧15 | 42 | 724.76±120.12 |  |  |  |  |  |

Title: Association between serum CCL-18 and IL-23 concentrations and disease progression of chronic obstructive pulmonary disease

Author list: Biaoxue Rong^1,2*^, Tian Fu^3^, Congxue Rong^4^, Wen Liu^5^, Kai Li^2^ & Hua Liu^6^

**Supp Table 2.** Relationship between clinical parameters and serum concentration of IL-23 in COPD patients (N﹦113). ^●^, twenty-one patients with AECOPD were unable to complete pulmonary function tests and did not participate in the statistical analysis of GOLD grade; ^▲^, the Levene’ s Test for Equality of Variances indicated that the variance was uneven, so the correction result of the t test was taken; ^◆^, "Yes" in the smoking category refers to current smokers. "No" refers to those who have never smoked or have quit smoking for more than 3 months. GOLD grade: 1﹦the forced expiratory volume in one second (FEV1) % predicted is more or equal to 80%, 2﹦the FEV1 % predicted is more or equal to 50%, but less than 80%, 3﹦the FEV1 % predicted is more or equal to 30%, but less than 50%, and 4﹦the FEV1 % predicted is less than 30%; mMRC, modified british medical research council for dyspnea scale for symptom classification of COPD. N, number; COPD, chronic obstructive pulmonary disease; AECOPD, acute exacerbation of COPD; IL-23, interleukin 23; M±SD, mean ± standard deviation; GOLD, Global Initiative for Chronic Obstructive Lung Disease; mMRC, modified british medical research council.

|  | Item | Correlation analysis | | | | |
| --- | --- | --- | --- | --- | --- | --- |
|  |  | CCL-18 and  IL-23 | CCL-18 and  FEV1/FVC | CCL-18 and  FEV1% Predicted | IL-23 and  FEV1/FVC | IL-23 and  FEV1% Predicted |
| Pearson correlation | Correlation coefficient | 0.780 | ﹣0.483 | ﹣0.502 | ﹣0.421 | ﹣0.536 |
|  | P value | <0.001 | <0.001 | <0.001 | <0.001 | <0.001 |
| ANOVA | Mean square | 1552.37 | 0.423 | 0.894 | 0.320 | 0.808 |
|  | F value | 172.077 | 27.113 | 29.972 | 19.129 | 26.23 |
|  | P value | <0.001 | <0.001 | <0.001 | <0.001 | <0.001 |
| Model summary | R square | 0.608 | 0.234 | 0.252 | 0.177 | 0.228 |
|  | Std. Error | 39.40 | 0.125 | 0.173 | 0.129 | 0.176 |
| Regression coefficient | Standardized coefficients | 0.78 | ﹣0.483 | <0.001 | ﹣0.421 | ﹣0.477 |
|  | T value | 13.118 | ﹣0.508 | ﹣5.465 | ﹣4.374 | ﹣5.121 |
|  | P value | <0.001 | <0.001 | <0.001 | <0.001 | <0.001 |
|  | 95% confidence interval | 0.379 to 0.514 | ﹣0.002 to ﹣0.001 | ﹣0.002 to ﹣0.001 | ﹣0.001 to ﹣0.000 | ﹣0.001 to ﹣0.0001 |
| Correlation model | Equation | *^Y﹦﹣84.706+0.698X* | *^Y﹦0.718*﹣*0.001X* | *^Y﹦0.698*﹣*0.001X* | *^Y﹦0.834*﹣*0.001X* | *^Y﹦1.048*﹣*0.001X* |

Title: Association between serum CCL-18 and IL-23 concentrations and disease progression of chronic obstructive pulmonary disease

Author list: Biaoxue Rong^1,2*^, Tian Fu^3^, Congxue Rong^4^, Wen Liu^5^, Kai Li^2^ & Hua Liu^6^

**Supp Table 3.** Correlation between CCL-18 and IL-32 concentrations and pulmonary function in COPD patients (n﹦92). CCL-18, chemokine (C-C Motif) ligand 18; IL-32, interleukin-32; FEV1, the value of forced expiratory volume in one second; FVC, forced vital capacity; ANOVA, variance analysis.

| ROC curve analysis of serum CCL-18 | | | | | | |
| --- | --- | --- | --- | --- | --- | --- |
| Criterion | Sensitivity | 95% CI | Specificity | 95% CI | +LR | -LR |
| >72 | 100.00 | 96.8 - 100.0 | 12.50 | 6.2 - 21.8 | 1.14 | 0.00 |
| >93 | 96.46 | 91.2 - 99.0 | 18.75 | 10.9 - 29.0 | 1.19 | 0.19 |
| >105 | 95.58 | 90.0 - 98.5 | 37.50 | 26.9 - 49.0 | 1.53 | 0.12 |
| >116 | 90.27 | 83.2 - 95.0 | 47.50 | 36.2 - 59.0 | 1.72 | 0.20 |
| >127 | 84.96 | 77.0 - 91.0 | 56.25 | 44.7 - 67.3 | 1.94 | 0.27 |
| >158 | 80.53 | 72.0 - 87.4 | 83.75 | 73.8 - 91.1 | 4.96 | 0.23 |
| >168.3* | 72.57 | 63.4 - 80.5 | 92.50 | 84.4 - 97.2 | 9.68 | 0.30 |
| >177 | 68.14 | 58.7 - 76.6 | 92.50 | 84.4 - 97.2 | 9.09 | 0.34 |
| >178 | 68.14 | 58.7 - 76.6 | 96.25 | 89.4 - 99.2 | 18.17 | 0.33 |
| >193 | 61.06 | 51.4 - 70.1 | 96.25 | 89.4 - 99.2 | 16.28 | 0.40 |
| >196 | 61.06 | 51.4 - 70.1 | 98.75 | 93.2 - 100.0 | 48.85 | 0.39 |
| >245 | 33.63 | 25.0 - 43.1 | 98.75 | 93.2 - 100.0 | 26.90 | 0.67 |
| >248 | 33.63 | 25.0 - 43.1 | 100.00 | 95.5 - 100.0 |  | 0.66 |
| ROC curve analysis of serum IL-23 | | | | | | |
| Criterion | Sensitivity | 95% CI | Specificity | 95% CI | +LR | -LR |
| >387 | 100.00 | 96.0 - 100.0 | 8.75 | 3.6 - 17.2 | 1.10 | 0.00 |
| >470 | 91.21 | 83.4 - 96.1 | 17.50 | 9.9 - 27.6 | 1.11 | 0.50 |
| >512 | 82.42 | 73.0 - 89.6 | 46.25 | 35.0 - 57.8 | 1.53 | 0.38 |
| >525 | 79.12 | 69.3 - 86.9 | 57.50 | 45.9 - 68.5 | 1.86 | 0.36 |
| >529 | 78.02 | 68.1 - 86.0 | 58.75 | 47.2 - 69.6 | 1.89 | 0.37 |
| >553 | 76.92 | 66.9 - 85.1 | 67.50 | 56.1 - 77.6 | 2.37 | 0.34 |
| >563.3** | 76.92 | 66.9 - 85.1 | 77.50 | 66.8 - 86.1 | 3.42 | 0.30 |
| >591 | 69.23 | 58.7 - 78.5 | 82.50 | 72.4 - 90.1 | 3.96 | 0.37 |
| >637 | 61.54 | 50.8 - 71.6 | 86.25 | 76.7 - 92.9 | 4.48 | 0.45 |
| >658 | 56.04 | 45.2 - 66.4 | 91.25 | 82.8 - 96.4 | 6.41 | 0.48 |
| >667 | 52.75 | 42.0 - 63.3 | 93.75 | 86.0 - 97.9 | 8.44 | 0.50 |
| >679 | 43.96 | 33.6 - 54.8 | 97.50 | 91.3 - 99.7 | 17.58 | 0.57 |
| >682 | 42.86 | 32.5 - 53.7 | 100.00 | 95.5 - 100.0 |  | 0.57 |

Title: Association between serum CCL-18 and IL-23 concentrations and disease progression of chronic obstructive pulmonary disease

Author list: Biaoxue Rong^1,2*^, Tian Fu^3^, Congxue Rong^4^, Wen Liu^5^, Kai Li^2^ & Hua Liu^6^

**Supp Table 4.** ROC curve analysis of serum CCL-18 and IL-32 concentration for distinguishing COPD from healthy people. *, cutoff values of CCL-18 to differentiate COPD from healthy people (AUC=0.870); **, cutoff values of IL -23 to differentiate COPD from healthy people (AUC=0.799). ROC, receiver operating characteristic curve; AUC, area under the ROC curve; CCL-18, chemokine (C-C Motif) ligand 18; IL-23, interleukin-23; COPD, chronic obstructive pulmonary disease; 95% CI, 95% confidence; +LR, positive likelihood ratio; -LR, negative likelihood ratio.

| Backward  deletion | Variables | Variables in the Equation for CCL-18 | | | | | | | |
| --- | --- | --- | --- | --- | --- | --- | --- | --- | --- |
|  |  | Regression  coefficients | S.E. | Wald | Df | *p* value | OR value | 95% C.I.for OR | |
|  |  |  |  |  |  |  |  | Lower | Upper |
| Step 1^a^ | Gender | 1.202 | 0.971 | 1.534 | 1 | 0.216 | 3.328 | 0.496 | 22.322 |
|  | Age | 1.056 | 0.692 | 2.328 | 1 | 0.127 | 2.874 | 0.740 | 11.155 |
|  | Smoke | 0.293 | 0.973 | 0.091 | 1 | 0.763 | 1.341 | 0.199 | 9.027 |
|  | GOLD grade | 0.819 | 0.505 | 2.628 | 1 | 0.105 | 2.268 | 0.843 | 6.103 |
|  | mMRC | 0.281 | 0.582 | 0.234 | 1 | 0.629 | 1.325 | 0.424 | 4.142 |
|  | Medical history | 1.771 | 1.200 | 2.179 | 1 | 0.140 | 5.877 | 0.560 | 61.732 |
|  | Constant | -5.500 | 1.863 | 8.717 | 1 | 0.003 | 0.004 |  |  |
| Step 2^a^ | Gender | 0.995 | 0.683 | 2.122 | 1 | 0.145 | 2.704 | 0.709 | 10.310 |
|  | Age | 1.122 | 0.661 | 2.886 | 1 | 0.089 | 3.072 | 0.842 | 11.216 |
|  | GOLD grade | 0.829 | 0.502 | 2.729 | 1 | 0.099 | 2.291 | 0.857 | 6.125 |
|  | mMRC | 0.245 | 0.567 | 0.187 | 1 | 0.665 | 1.278 | 0.421 | 3.882 |
|  | Medical history | 1.746 | 1.195 | 2.135 | 1 | 0.144 | 5.729 | 0.551 | 59.572 |
|  | Constant | -5.205 | 1.570 | 10.994 | 1 | 0.001 | 0.005 |  |  |
| Step 3^a^ | Gender | 0.922 | 0.657 | 1.972 | 1 | 0.160 | 2.515 | 0.694 | 9.111 |
|  | Age | 1.079 | 0.649 | 2.761 | 1 | 0.097 | 2.942 | 0.824 | 10.505 |
|  | GOLD grade | 0.962 | 0.396 | 5.891 | 1 | 0.015 | 2.618 | 1.203 | 5.694 |
|  | Medical history | 1.835 | 1.175 | 2.442 | 1 | 0.118 | 6.268 | 0.627 | 62.660 |
|  | Constant | -4.857 | 1.333 | 13.266 | 1 | 0.000 | 0.008 |  |  |
| Step 4^a^ | Age | 0.650 | 0.550 | 1.394 | 1 | 0.238 | 1.916 | 0.651 | 5.635 |
|  | GOLD grade | 0.914 | 0.390 | 5.493 | 1 | 0.019 | 2.493 | 1.161 | 5.352 |
|  | Medical history | 2.000 | 1.154 | 3.003 | 1 | 0.083 | 7.391 | 0.769 | 70.991 |
|  | Constant | -4.299 | 1.232 | 12.180 | 1 | 0.000 | 0.014 |  |  |
| Step 5 ^a^ | GOLD grade | 1.017 | 0.372 | 7.479 | 1 | 0.006 | 2.764 | 1.334 | 5.728 |
|  | Medical history | 2.009 | 1.150 | 3.055 | 1 | 0.008 | 7.358 | 0.784 | 70.971 |
|  | Constant^★^ | -4.193 | 1.213 | 11.952 | 1 | 0.001 | 0.015 |  |  |

Title: Association between serum CCL-18 and IL-23 concentrations and disease progression of chronic obstructive pulmonary disease

Author list: Biaoxue Rong^1,2*^, Tian Fu^3^, Congxue Rong^4^, Wen Liu^5^, Kai Li^2^ & Hua Liu^6^

**Supp Table 5.** Logistc regression between serum CCL-18 concentrations and clinical parameters of COPD. ^a^, variable(s) entered on step 1: gender, age, smoke, GOLD grade, mMRC, medical history; ^★^, Logistc Regression Equation: P=1/[1+e-(-4.193+1.017GOLD grade+2.009medical history)]. COPD, chronic obstructive pulmonary disease; CCL-18, chemokine (C-C Motif) ligand 18; S.E., standard error of regression coefficient; GOLD, Global Initiative for Chronic Obstructive Lung Disease; mMRC, modified british medical research council; Wald, test statistics for regression coefficients; Df, degrees of freedom; OR, odds ratio; 95% C.I., 95% confidence interval.

| Backward  deletion | Variables | Variables in the Equation for IL-23 | | | | | | | |
| --- | --- | --- | --- | --- | --- | --- | --- | --- | --- |
|  |  | Regression  coefficients | S.E. | Wald | Df | p value | OR value | 95% C.I.for OR | |
|  |  |  |  |  |  |  |  | Lower | Upper |
| Step 1^a^ | Gender | 0.122 | 1.053 | 0.013 | 1 | 0.908 | 1.130 | 0.143 | 8.900 |
|  | Age | -0.567 | 0.713 | 0.634 | 1 | 0.426 | 0.567 | 0.140 | 2.292 |
|  | Smoke | 0.258 | 1.047 | 0.061 | 1 | 0.805 | 1.294 | 0.166 | 10.064 |
|  | GOLD grade | 1.292 | 0.590 | 4.799 | 1 | 0.028 | 3.640 | 1.146 | 11.564 |
|  | mMRC | 0.177 | 0.614 | 0.083 | 1 | 0.773 | 1.194 | 0.358 | 3.976 |
|  | Medical history | 0.859 | 1.285 | 0.447 | 1 | 0.504 | 2.361 | 0.190 | 29.300 |
|  | Constant | -3.147 | 1.793 | 3.079 | 1 | 0.079 | 0.043 |  |  |
| Step 2^a^ | Age | -0.577 | 0.709 | 0.662 | 1 | 0.416 | 0.562 | 0.140 | 2.254 |
|  | Smoke | 0.163 | 0.651 | 0.063 | 1 | 0.802 | 1.177 | 0.328 | 4.218 |
|  | GOLD grade | 1.299 | 0.586 | 4.913 | 1 | 0.027 | 3.667 | 1.162 | 11.568 |
|  | mMRC | 0.160 | 0.596 | 0.072 | 1 | 0.788 | 1.174 | 0.365 | 3.774 |
|  | Medical history | 0.868 | 1.283 | 0.458 | 1 | 0.498 | 2.383 | 0.193 | 29.446 |
|  | Constant | -3.023 | 1.435 | 4.437 | 1 | 0.035 | 0.049 |  |  |
| Step 3^a^ | Age | -0.504 | 0.648 | 0.605 | 1 | 0.437 | 0.604 | 0.170 | 2.150 |
|  | GOLD grade | 1.296 | 0.586 | 4.896 | 1 | 0.027 | 3.656 | 1.160 | 11.527 |
|  | mMRC | 0.168 | 0.594 | 0.080 | 1 | 0.777 | 1.183 | 0.369 | 3.788 |
|  | Medical history | 0.798 | 1.254 | 0.405 | 1 | 0.525 | 2.221 | 0.190 | 25.960 |
|  | Constant | -2.926 | 1.381 | 4.489 | 1 | 0.034 | 0.054 |  |  |
| Step 4^a^ | Age | -0.498 | 0.645 | 0.597 | 1 | 0.440 | 0.607 | 0.172 | 2.150 |
|  | GOLD grade | 1.390 | 0.486 | 8.187 | 1 | 0.004 | 4.013 | 1.549 | 10.397 |
|  | Medical history | 0.864 | 1.230 | 0.493 | 1 | 0.483 | 2.372 | 0.213 | 26.443 |
|  | Constant | -2.738 | 1.210 | 5.126 | 1 | 0.024 | 0.065 |  |  |
| Step 5^a^ | Age | -0.536 | 0.655 | 0.670 | 1 | 0.413 | 0.585 | 0.162 | 2.111 |
|  | GOLD grade | 1.589 | 0.423 | 14.090 | 1 | 0.000 | 4.898 | 2.137 | 11.229 |
|  | Constant | -2.174 | 0.820 | 7.025 | 1 | 0.008 | 0.114 |  |  |
| Step 6^a^ | GOLD grade | 1.439 | 0.367 | 15.352 | 1 | 0.000 | 4.215 | 2.052 | 8.656 |
|  | Constant | -2.153 | 0.819 | 6.919 | 1 | 0.009 | 0.116 |  |  |

Title: Association between serum CCL-18 and IL-23 concentrations and disease progression of chronic obstructive pulmonary disease

Author list: Biaoxue Rong^1,2*^, Tian Fu^3^, Congxue Rong^4^, Wen Liu^5^, Kai Li^2^ & Hua Liu^6^

**Supp Table 6.** Logistc regression between serum IL-23 concentrations and clinical parameters of COPD. ^a^, variable(s) entered on step 1: gender, age, smoke, GOLD grade, mMRC, medical history; ^★^, Logistc Regression Equation: P=1/[1+e^-(-2.153+1.439GOLD grade)^]. COPD, chronic obstructive pulmonary disease; IL-23, interleukin-23; S.E., standard error of regression coefficient; GOLD, Global Initiative for Chronic Obstructive Lung Disease; mMRC, modified british medical research council; Wald, test statistics for regression coefficients; Df, degrees of freedom; OR, odds ratio; 95% C.I., 95% confidence interval.
